# Ethical underpinnings for the development of health literacy in schools: ethical premises (‘why’), orientations (‘what’) and tone (‘how’)

**DOI:** 10.1186/s12889-018-5224-0

**Published:** 2018-03-06

**Authors:** Leena Paakkari, Shanti George

**Affiliations:** 1 Research Center for Health Promotion, Faculty of Sport and Health Sciences, University of Jyväskylä, P.O.Box 35 (L), 40014, Jyväskylä, Finland; 2 The Hague, Netherlands

## Abstract

**Background:**

Schools are seen as crucial environments to influence and develop the health literacy of new generations, but without sufficient reflection on the ethical underpinnings of intentions and interventions around health literacy. In contrast, we argue here that ethics are fundamental to all education. The article adopts a ‘One world’ approach that generalizes broadly across the so-called Global North and Global South. It also generalizes across various age groups among school pupils, advocating age appropriate application of the arguments advanced.

**Main text:**

Our analysis examines why health literacy should be promoted in schools and argues that the purpose should embrace the values of social justice and should not stop at individual and national cost benefit analysis. Discussion about the orientation of health literacy highlights meta-cognitive skills around critical thinking, self-awareness and citizenship rather than lists of practical skills. Finally, approaches to health literacy in classrooms are presented with an ethical tone that draws attention to the power relations responsible for health inequities and that does not assume that such power relations are the given framework for health literacy interventions and activities. These arguments are reinforced by urging that related debates address dynamic social realities such as international migration.

**Conclusions:**

We reiterate the need for ethical questions to be consciously and systematically addressed from early on, beginning with intentions to promote health literacy even before these intentions are translated into action, within the political space where education meets public health and health promotion. We underline again the context of fluidity and dynamism, as new challenges emerge within pedagogies and curricula, especially in response to changing populations in the society around.

## Background

Children’s health and health literacy are rated as crucial in promoting public well-being [1], and schools are identified as important arenas for developing related health competences [1–4]. Increased interventions, measurements and research carried out among school pupils are not however accompanied by sufficient focus on the ethical aspects encompassed by health literacy promotion, even though strong reasons can be advanced for such a focus. Teaching is in general an ethically loaded profession [5, 6], and the teaching of health issues raises additional ethical questions [7]. Work with pupils who are at extremely impressionable ages generates ethical tensions [8]. School environments, this paper argues, in fact require more rather than less ethical reflection around some problematic aspects of health literacy.

For a decade and a half now, good health literacy has been identified as a key public health priority [9] that manifests favourable consequences at both individual and societal levels. It is linked to better interpretation of health knowledge and improved overall health [10] as well as lowered risk-taking [11]. Societal level outcomes include, for example, less hospitalization [10] and reduced health costs [12]. Improving health literacy within populations is therefore now an explicit public health goal in many policy papers in various countries, and in several global policy reports (e.g. [13]), thereby legitimizing attempts to influence individuals’ health literacy. Extensive effort has been expended to measure health literacy in order to inform politicians about current levels across population groups, including among school pupils.

Supporting people’s competence, at all stages of the life course, to promote and maintain personal and community health - i.e. health literacy - is vital [14, 15], yet the ethical justifications around intentions or actions to develop health literacy are insufficiently discussed. We therefore argue below that ethical difficulties should be recognized in all health promotion and health education practices (e.g. [16, 17]), and ethical considerations should be central in health-related decision making in order to avoid causing harm and injustice [18].

This paper raises and discusses some relevant ethical considerations. The three sections that follow discuss *why* health literacy should be encouraged among school pupils, *what* should be developed within the health literacy curriculum and *how* this should be carried out. The analysis will centre on ethical premises (‘why’), orientations (‘what’) and tone (‘how’), rather than on the practical ‘nuts and bolts’ of health literacy in schools. Within the constraints of space here, more attention will be paid to premises and orientations, and the ‘how’ of health literacy promotion in classrooms will be sketched in towards the end in order to provide a bridge to relevant pedagogical interactions.

We clarify at the outset that our discussion of the ‘ethical’ follows the perspectives provided by Alexander [19], that “ethics should be understood as first philosophy in education” (p. 1), and especially in “democratic education that would promote the rights of people with diverse visions of the good life to live side by side in a common civil society’” (p. 2).

This broad orientation to ethics in education is applied by us to a suitably broad global context, where – to extend Alexander’s words as just quoted – democratic education promotes the rights of people with diverse visions of the good life to live side by side in a common global civil society. Our discussion will override the distinction between Global North and Global South that is often reduced to a dichotomy between ‘developed/developing countries.’ We will instead adopt a One World approach that urges that Global North and Global South learn together and from each other in order to co-determine the future [20, 21]. The analysis below therefore ranges from the many national contexts where children are not assured of the right to schooling to other country contexts such as Finland where universal schooling includes a curriculum in health education – and we will look critically at both as well as at the spectrum in between. Limits of space however prevent a detailed citing of cases and our arguments therefore remain broad and general, anticipating discussions that will follow on from this one and that will illuminate specific cases.

The ethical purpose, orientation and tone of pedagogies around health literacy in schools have been framed here by the questions ‘Why?’, ‘What?’ and ‘How?’ respectively. We have to limit ourselves to these, although many other ethical questions can be brought to bear. The query ‘When?’ for example leads into analyses of different perspectives associated with age and maturity, in the context of school pupils as well as further on in the life course. In this paper we consider school pupils at the general level without any particular focus on certain age groups, once again anticipating future discussions of pupils at various specific ages. We extend Alexander’s [19] vision of “awareness of and ability to exercise age appropriate self-governance” (p. 13) to issues around taking responsibility for one’s own and other people’s health, and we note that ‘age appropriate’ extends to the evolving capacities [22, 23] of even very young children [24].

We would also like to argue that the ethical premises underpinning the development of *health* literacy in schools hold good for the development of literacy in general as well as of the various forms that we can cluster together under the heading of ‘literacies’, whether in the crucial environment of schools or more widely. Health literacy is therefore only one of the multiple literacies that enable people to enjoy freedom of choice and to experience empowerment through autonomy, as discussed in the section that follows. Developing any form of literacy among school pupils and within the general public is a moral act. All literacies tend to follow socio-economic hierarchies and to raise difficult questions – addressed in the section below – around ethics, equity and disparity.

## Ethical premises: Why should school pupils be encouraged to develop their health literacy?

As a starting point for ethical reflections about the purpose of developing health literacy in schools, we support Sen’s [25] argument that “health is among the most important conditions of human life and a critically significant constituent of human capabilities which we have reason to value” (p. 660). A deprivation in people’s health negatively influences their “capability for health functioning and agency” ([26], p. 999), and thereby threatens human flourishing, whereas improvement in health promotes and maintains relevant capability and human capital [27]. Conversely, human capabilities may increase good health or are part of the opportunities to do so, enabling individual freedom to choose certain levels of functioning [28], for example to eat healthily.

Viewing health literacy as a capability that enables individuals to enjoy freedom is closely related to empowerment as an ethical principle of health promotion (see [17]). Health literacy is often considered to be a critical factor in empowerment [4, 14, 29], as it enables greater independence in taking care of one’s health and thereby supports autonomy and freedom. From this standpoint, health literacy per se could be seen as everyone’s right, including school pupils. To refrain from teaching health issues would be ethically questionable, given the universal right to know about how to promote one’s own health and that of others, how to prevent and cure illness, and related issues [30]. The “mandate to assure and protect the health of the public is an inherently moral one” ([31], p. 317), and therefore developing health literacy among school pupils and the public more generally is a moral act, especially since the gap between the demands for taking care of one’s health and people’s actual skills in doing so is evident and growing ([13], see also [32]).

Notions of ‘good health’ are often translated into guidelines intended to influence people’s health choices. Usually such guidelines draw strongly on medical models about how people should live (see [33]), reflecting factual certainties and authority [34] and highlighting the importance of people behaving in a responsible manner and avoiding risky behaviour -- with the terms ‘being responsible’ and ‘acting riskily’ already expressing certain values and ways of behaving that people including school pupils should adopt or avoid [35]. Such guidelines capture a notion of what is considered good health and what should be pursued [36], and related ways of behaving that may be sanctioned or rewarded [35]. Questions about what is health and being healthy, who decides which ways of behaving are better than others, and whether it is a person’s responsibility to behave in a healthy manner, gain in importance.

Judgements and rankings of ‘healthy’ behaviour tend to correlate positively with socio-economic hierarchies (see [37]). The argument that “health literacy is an issue of ethics and equity and is essential to reducing disparities” ([32] p. 151), provides an entry point for our discussion to examine the complex relationship between ethics, equity and disparities in the context of schools and health literacy.

One of the main purposes of education is to reduce inequalities [38]. If education per se “improves the overall health and well-being of learners” ([38], p. 48), acquiring health literacy skills at school could decrease health inequalities between children from different socio-economic backgrounds [32]. Would this decrease be guaranteed if all children learned these skills in schools in an equal way? Unfortunately the issue is not so simple.

A huge initial challenge remains how to guarantee education in general for every child. Despite positive progress in widespread access to basic education during the last 15 years, more than 1.7 million children worldwide are not in school [39]. The ethical consideration that education per se is a universal right underpins all ethical reflections about health literacy as part of school education. General literacy skills are a fundamental right of every human being, including as a foundation for various health literacy competencies [40], although we do not focus on literacy skills in this paper.

Further, “the gap in learning outcomes between rich and poor - within and between countries - is high and often growing” ([38], p. 9). Unequal investments in children and their health is one of the main causes for health disparities within and across countries [41]. Schools differ in resources, thereby rendering unequal their pupils’ chances to learn all currently needed skills. Where privileged parents are able to choose schools for their children, they opt for so-called superior schools that have better trained teachers and enhanced resources [42]. Schools vary in their ability to respond to new demands, for example in their access to information technology, even though technology “can play a central role in developing skills needed in the 21st century and improving access to lifelong learning opportunities” ([38], p. 14). Rapid technological developments -- related to constantly increasing access to and amounts of online health information -- can increase disparities further.

What is offered in schools is therefore crucial [42], and if health literacy is part of the curriculum only in some countries, states or schools, the unequal chances for pupils to learn health competencies may well increase the gap between pupils with low and high levels of health literacy. Following the earlier association between improved health literacy and better health outcomes, school education may paradoxically promote health inequalities. From the ethical viewpoint, we note that unequal education probably increases health inequalities.

All the same, since education does promote individual health and wellbeing, and can contribute to greater empowerment within society, it could also be argued that developing health literacy through educational practice in all schools is inevitably good. Indeed, Downey, Hippel and Broh [43] found that schooling does reduce cognitive inequalities across socio-economic status and compensates for limited learning experiences outside school. Although the cognitive gap between various groups “does not close in school, it does not widen as fast as it otherwise might” ([43], p. 632; see also [44]). In contrast, the different environments outside school – family, friends and neighbourhood -- appear to influence widening cognitive gaps between various groups of children [43].

Thus one ethical response to the WHY question – the *purpose* of promoting the learning of health literacy in schools – is that this can help to reduce socio-economic inequalities. Establishing school-based learning standards for health literacy could contribute to tackling health disparities [32]. Again from an ethical viewpoint, we note that learning health literacy competencies in school can help to narrow gaps and reduce disparities.

Is it fair, we may now ask, to educate pupils who already have high levels of health literacy – derived for example from their privileged non-schooling experiences -- if education does widen the gap? The argument that it is both right and more cost effective to focus on children most in need [45] seems irrefutable, following ethical principles of equity to narrow the gap in health outcomes among and within various population groups and to recognize that actions which target vulnerable and disadvantaged people are needed [17], and should be prioritized [26]. Given arguments however, about health gaps between women and men, that “it would be morally unacceptable to suggest that women should receive worse health care than men so that the inequality in the achievement of health and longevity disappears” ([25], p. 661), it can similarly be argued that it is immoral not to promote the health literacy of those pupils who already have higher levels. As education in general is everyone’s right [46], so is education on health issues: “the universal right of access to health literacy should be recognized” ([13], p. 23), along with the need for targeted interventions. A special focus on marginalized groups can yield positive outcomes that benefit everyone in society [39], at the same time that it is ethically justified to maintain existing high health literacy among certain population groups.

Current debates emphasize the increased numbers of immigrants that raise new challenges for schools in securing all pupils’ well-being [47]. Rapid and steady integration of immigrant children is needed to avoid further marginalization [39], by taking special measures [48], since every citizen has the right to learn skills required in a particular society, and the purpose of education is to guarantee this.

Widespread perceptions that immigrants represent deficits that need to be rectified – especially low income immigrants in rich countries from other parts of the world -- should also be questioned on ethical grounds [33]. Indeed, perceptions that low income groups in general represent deficits to be redressed should be simultaneously confronted. Arguments quoted earlier about ‘cognitive gaps’ and ‘cognitive inequalities’ derive from measurements along accepted social hierarchies wherein educated upper class lifestyles provide the norm, thereby privileging these lifestyles over others. Ethical principles question such representation of elite lifeways as the epitome of health within a society. ‘Rich in income and status’ does not necessarily mean ‘rich in all ways’ or right in all health practices.

Hayward’s [49] insightful research among primary school children in New Zealand did not automatically correlate well-being with income or status. Instead, her varied sample of schools highlighted the different assets and forms of well-being enjoyed by diverse categories of children.

- Children in high income, largely ‘Anglo’ urban neighbourhoods benefitted from structured activities under privileged circumstances, yet these children wished for less structure and more informality in daily timetables. They lived in affluent ‘leafy’ suburbs, but seemed somewhat divorced from their natural environments.
- In schools in low to middle income urban communities, children described “comparatively thick and dense networks of social interaction. These children listed a huge range of formal and informal clubs and activities… [and] spoke about interacting regularly with parents, siblings, cousins, friends, the parents of friends, grandparents and older and younger associates that they knew well in a neighbourhood that defined their primary ecological world” ([49], p. 90). This included single parent female-headed households, notably Maori (the indigenous people of New Zealand).
- Children at rural schools enjoyed expansive outdoor play and various informal activities in more natural settings. They belonged to fewer formal clubs than city counterparts but played more sport. These children used language in more nuanced ways, especially if their home language was Maori, when describing the zoological, botanical and social landscape.

Hayward’s research suggests that we must move between children’s varied perspectives in today’s plural multi-ethnic societies, rather than viewing each society as some homogenous cultural world wherein certain health practices fit everyone. Instead, different cultural realities interpenetrate in globalized societies. Ethics guide us away from assuming that rich countries have all the cultural answers about health and towards respect for the new knowledge that immigrants can bring with them.

Juxtaposing cultural perspectives from across the globe allows exploration of other cultural worlds and different notions of health developed by diverse cultures. Decentred deliberations [50] denote “public conversations that cross local, regional and national spaces, and across time” ([49], p. 128). Respectful conversations within multicultural societies will enable us to gain a better grip on complex issues around diversity [51], including in the context of health literacy.

These arguments hold good in a world of continuing migrations in different directions: “education must prepare learners to live and work abroad” ([38], p. 13). Individual levels of health literacy depend on the demands of the current environment. School education should therefore be adapted to meet the expectations of pupils from varying backgrounds and should enable all pupils to cope with new situations and different demands relevant to health care.

To summarize, why then should health literacy be cultivated in schools? Health and education are universal rights – and thus health education doubly so -- and schooling that spans diverse social groups can reduce disparities. At the same time, tensions exist between the need and potential for health literacy promotion in educational settings to address health inequalities within society, and the reality that schools can augment inequalities rather than address them. This tension will be explored further in the following sections, where we will argue that ‘what’ the health literacy curriculum is oriented towards and ‘how’ health literacy is addressed can constitute the difference between whether schools exacerbate health inequalities or reduce them.

## Ethical orientations: What should be emphasized within health literacy curricula?

Current heated debates -- on what children should learn to contribute to their well-being – perpetuate a long tradition. Nussbaum [52] describes the ancient Greeks as contrasting two kinds of education, (1) a “highly disciplined patriotic regimen, with lots of memorization and not much room for questioning” (p. 1) and (2) the ‘Think-Academy’ run by Socrates, where a youth “will learn to think critically about the social origins of apparently timeless norms… [and] will learn to construct arguments on his own” (p. 1). The latter was seen as extremely threatening – an opinion that continues to be held today, especially in the context of norms around health.

We argue here that health literacy competencies in schools should *not* denote ‘some highly disciplined’ medicalized ‘regimen, with lots of memorization’ – for example about ingesting precise amounts of particular nutrients – and ‘not much room for questioning.’ Following Apple [53], “what our children are to know and the values this should embody is serious business” (p. 1). We therefore adopt values -- and especially the ethical principles highlighted above – to guide assessment of what schools should cover under the rubric of health literacy, since health education like all education is a moral act that should enable people to function well in the world. Apple questions definitions of ‘good’ students as those whose knowledge is ‘good’ enough to secure ‘good’ jobs, and urges that ‘good’ be used in the moral sense when referring both to individuals and societies. Schools and teachers succeed in facilitating good education when health literacy is “seen as an asset to be built, as an outcome to health education and communication that supports greater empowerment in health decision-making” ([40], p. 2074).

The ongoing health literacy crisis [13] is not caused by lack of information. Information abounds in much of today’s world – including for adolescents and children – as a consequence of globalization and the technology that supports rapid development of the internet and media [54]. Children make use of the internet at increasingly younger ages, with a recent study in Finland revealing that 81% of children aged seven to eight are allowed to use the internet at home, and one third does so daily [55].

Neither is the crisis about skills required to gain access to information, as was the case even in the late twentieth century when information was limited, not easily available and took different forms. Now, in contrast, the internet’s exponential development in a globalized world requires skills to use effectively an abundance of health information and to distinguish between information of varying quality through comparison, classification and assessment of credibility [56]. When a mismatch is identified between social demands and the skills individuals now require to take care of their health [13], such complex skills are what we must recognize in an environment of complicated challenges. Many current health-related issues are so-called wicked problems -- unstable, unpredictable and in a nonlinear relationship with their causes and effects [1]. Indeed, the full context would include many more challenges, for example the tensions between environmental sustainability and economic growth [54].

Against this backdrop, we ask not only about skills and competencies within health literacy but how these are *valued*. Difficult decisions about ‘the right skills’ involve value judgements when low or high levels of health literacy – and insufficient and sufficient levels – are assessed. Measurement should make explicit what it is that we measure when we are measuring health literacy, and why.

To the question, where does health literacy end, we would answer that it ends when someone – a school pupil in this case -- moves from learning outcomes to grasping the probable consequences of these outcomes for her or his personal characteristics, behaviour and overall health within the wider social context. Should motivation or attitudes be included in health literacy components, since these have been emphasized as part of the key competencies of citizens in general [54]? Can it be said that someone’s levels of health literacy are low if she or he chooses differently from the suggested guideline after critical reflection? And to what extent are we able to take into account unequal chances of health, given extensive research that shows how various environmental factors influence people’s health and health choices (e.g. [57])? Obesity, for instance, cannot be addressed only by individual behavioural changes, considering the role that obesogenic environments play in creating life surroundings and conditions conducive to obesity [58]. If societal processes expose people to health damaging conditions [1], which affect their attitudes and motivation as well as their health behaviour, should we really say that to be seen as health literate “individuals *can* and *should* exert fundamental control over their health through careful and rational avoidance of risks” ([59], p. 68), thereby denying environmental influences and placing the locus of responsibility on individuals alone?

Similarly, is it ethically right to *emphasize* in all contexts and among all population groups that abilities should be developed -- under ‘health literacy’ -- to assess various alternatives related to medical treatment, and related risks and benefits, when in some (or even many) real life contexts the people concerned may have only one option [9], because of limited spending power or other constraints? Since contextual issues critically determine what is needed to manage health well, we question whether some standard list of skills can do justice to the realities around health literacy. Such lists seem more oriented to hypothetical thinking about skills that *might* be needed in future and situations that *may* arise later in life, rather than real people’s struggles in the here and the now.

We follow calls for a focus on broader competence areas instead of narrow skills [54], despite extensive literature on the essential skills of twenty first century citizens that include various lists (e.g. [56]). The OECD suggests that a few select universal key competencies should provide the focus in schools, and we support this in the context of health literacy. To be seen as a key competence, the behaviour required should “contribute to valued outcomes for societies and individuals; help individuals meet important demands in a wide variety of contexts; and be important not just for specialists but for all individuals” ([54], p. 4).

Therefore, despite the challenges related to identifying lists of skills, we agree on some universal learning goals that meet the criteria for key competencies as defined above by the OECD. We maintain that it is ethically vital to encourage certain competencies among school pupils – notably critical thinking, self-awareness and citizenship (see [3]) – and ethically questionable to neglect them. In contemporary school contexts, such competencies will support autonomous learning and agency, critical attitudes and responsible citizenship.

Development of *critical thinking* helps guard against the accumulation of socio-political power in a few hands [60], and enables evaluation of how various ‘health needs’ “have been constructed, manipulated and perhaps obfuscated by the interests of the ‘health industry’ ”([34], p. 98). Such questioning of authority serves as a countervailing force against indoctrination (described by Hanks [61] as the cultivation of certain belief, values and skills regardless of one’s own motives). Socialization, defined as the insertion of pupils into existing social, cultural and political orders [62], becomes evident in health literacy discussions that highlight health as a universal value although it is defined in very narrow terms, and pupils as active citizens who should contribute to raising health literacy levels by adopting particular health behaviours. Given prevailing prescriptions of ‘good health practices’ that people must recognize and adopt universally, critical thinking stimulates school pupils to examine what ‘good’ implies in a specific context and who has defined it and to what purpose:

“Critical thinking requires the attitude that knowledge is not fixed but always subject to re-examination and change… the attitude that there is no question which cannot, or should not, be asked… an awareness of, and an empathy for, alternative world views… a tolerance for ambiguity… an appreciation for alternative ways of knowing… a sceptical attitude towards text… [and] a sense of the complexity of human issues” ([63], p. 55-64).

The development of *self-awareness* -- through reflective and reflexive abilities in general, and as learners [3] – stimulates pupils to become aware of their *own* wishes, preferences, values and attitudes, to find their own voice, and to locate each one’s position in the world [64]. Such ‘thinking for oneself’ relates to autonomy [61] as well as independence of the existing orders [62], and supports freedom from passive following of traditions [65]. The related concept of individuation or subjectification [66] is closely related to “emancipation, that is, towards ways of doing and being that do not simply accept the given order but have an orientation toward the change of the existing order so that different ways of doing and being become possible” (p. 64). Such ‘practical reason’ is rooted in “being able to form a conception of the good and to engage in critical reflection about the planning of one’s life” ([67], p. 41). Self-awareness can also help to withstand the impact on identity formation of media environments in various forms, impact that is exemplified by a meta-analysis conducted by Grabe, Ward and Hyde [68] which revealed that media exposure is associated with negative body image and eating disorders among women of all ages.

*Citizenship* as a vital component of health literacy is closely related to critical thinking and self-awareness in supporting pupils to find their own way of being part of the society they belong to and to contribute to it, and not to adopt uncritically various values and practices that are pressed on them by the authorities or by peers. Citizenship education may have elements of indoctrination if possibilities are narrowed and limited, and ideas and beliefs are accepted unquestioningly. This is particularly important now that societies are more heterogeneous than they once were. One’s own cultural context should not be seen as the norm [69]. Pupils should be encouraged to understand and respect difference, and to acknowledge difference in themselves [19], as well as for example among immigrant pupils. Citizenship as a component of health literacy highlights children as ‘being’ active citizens in their own right, rather than only as ‘becoming’ adults and future members of society (see [70, 71]), just as the development of health literacy contributes to current well-*being* in addition to future well-*becoming* (see [72]). Children’s rights to be respected, heard and involved in the decisions that influence their lives [73] are thereby supported.

Citizenship thus refers to abilities to act in an ethically responsible way and to take social responsibility [3], by identifying and influencing the factors that contribute to collective health within the world at large [74]. Citizenship relates to participation as an ethical principle of health promotion [17], providing opportunities for people to promote and protect their own and each other’s health and well-being [1].

All three competences call for meta-cognitive skills that are essential for lifelong learning. Given that “no one is ever fully health literate” ([13], p. 8) and that health literacy depends on current challenges, it is both essential and ethical that school pupils be encouraged to develop these meta-cognitive skills.

The previous section argued why the emphases within health literacy curricula can influence whether schools exacerbate or reduce socio-economic inequalities. The present section has elaborated on this by suggesting that a narrow focus on checklists of health related skills is part of an education that maintains the world as it is with all its unfair disparities, whereas an ethically sensitive orientation towards a few crucial broad competencies – such as our examples of critical thinking, self-awareness and citizenship – can provide a pathway towards the world as it might be [20]. Critical thinking among individuals and across society locates health information and prescription within the context of socio-political realities. Self-awareness precludes automatic acceptance of ‘authoritative’ (and often authoritarian) decisions about health and encourages scrutiny of the processes at work and the consequences for oneself as well as for relevant others. Citizenship should identify all members of society as these relevant others and -- rather than comply obediently with established wisdom -- should examine individual and social costs and benefits, most especially among marginalized population groups. Health literacy along these lines would be embedded in socio-political and socio-economic literacy that goes beyond reading the words on medical packaging to reading the world around that is riddled with inequalities – inequalities that should be made transparent in public debates about health and that must be confronted through these debates. Such health literacy would exemplify ‘a situated ethics of social justice’ ([75], p. 17). How exactly such a ‘situated ethic’ can be articulated within schools and classrooms is the subject of the section that follows.

## Ethical tone: How should health literacy be addressed in classrooms?

Our opening discussion on the purpose of promoting health literacy in schools emphasized the values of social justice over narrow cost benefit considerations. Our subsequent analysis of the form that health literacy should take among school pupils highlighted meta-cognitive orientations towards critical thinking, self-awareness and citizenship rather than lists of highly specific skills. On similar lines, this closing examination of *how* health literacy should be fostered in schools focuses on the broader ethical tone of activities and not the activities themselves. It is necessarily the shortest of the three sections, given constraints of space, and will suffice only to sketch *how* classroom activities can embody and ground meta-cognitive orientations and values associated with social justice.

The familiar image of an iceberg can be usefully invoked here, with the visible activities (the ‘how’) of health literacy education resting on the unseen foundation of values associated with the purpose (‘why’) and orientation (‘what’) of such education. Clearly health literacy activities generated by the broad values just discussed will differ radically from health activities based on limited skill sets and cost benefit.

Our first argument here is that ethically oriented health literacy is embedded in ethically sensitive education more generally and must therefore be grounded in what Haynes [76] describes as ‘the ethical school’: “Ethics works most successfully in an open community of inquiry in which each of the participants has an equal voice” (p. 39). Classroom discussions of health literacy cannot maintain an ethical tone if such a tone is more or less lacking in the general school environment, although exchanges about health can provide a starting point for struggle towards wider ethical sensitivity in school communities. Fisher [77] provides an illuminating illustration of how the explicit addressing by teachers and pupils of concepts related to ethics and social responsibility offers “a necessary first step towards facilitating students’ critical engagement” (p. 400).

Haynes’ description above of an ethical school as ‘an open community of inquiry’ rather than some closed community of compliance makes it clear that the ‘how’ of health literacy does *not* involve universal prescriptions of what people should eat and how they should exercise, but that these subjects should instead be open to critical thinking and shared inquiry:

“Freedom of information is essential to freedom of thought. We need freedom of speech not simply to emphasize the importance of but to ensure free access to different ways of describing the world. If we destroy the possibility of personhood and autonomy, we preclude the possibility of free choice on which knowledge is dependent” ([76], p. 131).

In matters of health, as with religion, Haynes maintains that “the good life must be freely chosen” ([76], p. 133). We advocate neither ‘unlimited freedom’ nor ‘a degree of constraint that demands ‘one right way’ ([78], p. 210). Instead, “the twin issues of freedom and discipline” ([78], p. 123) are spanned through a kind of “disciplined freedom”‘([78], p. 125, as applied by [79]).

Such freedom must be placed within the context of power relations: “one end of ethics is to share power, or at least to guard against the abuse of power” ([76], p. 49). We will concentrate on power relations as expressed in (a) approaches to knowledge, (b) teachers’ interactions with pupils and (c) pupils’ relationships with each other. In all three cases we will start with what is *not* an ethical tone, in order to highlight how current hegemonies find expression in standard discourses within health literacy. Strong emphasis is placed on the role of the classroom teacher, so we duly acknowledge here the (typically) neo-liberal, performative, audit culture within which many teachers today must struggle to conduct their work.

Knowledge about health is not inscribed on stone tablets to be unveiled by health education teachers and then dutifully memorized by compliant pupils. An ethical tone stimulates awareness that knowledge is usually complex and uncertain and rarely simple and definite, requiring ‘pedagogies of uncertainty’ [80] – which is why an orientation towards critical thinking and individual reflection was emphasized in the previous section. “‘Participatory decision-making’… assumes professionality and personal autonomy, the possibility of thinking for oneself” ([76], p. 36).

Health education teachers should not be guardians and propagators of what Biesta [62] describes as the existing social, cultural and political orders. Instead, teachers must themselves be critically aware of how power relations construct and transmit established social wisdom, as well as the role that the teaching profession can play in entrenching conservative knowledge. A teacher is more likely to offer pupils a genuine opportunity to develop critical thinking and reflection skills if there is realization that he or she should not manipulate classroom debate by selecting certain issues as the ‘right’ ones and by emphasizing certain materials and not others [7].

Diverse identities, backgrounds and experiences among pupils within a classroom do not constitute a threat to health literacy, but instead are an asset to be built on as usefully providing ‘different ways of describing the world’ ([76], p. 131)*.* Plurality should be “seen as that which makes our being with others possible and real in the first place” ([81], p. 92), thereby fostering the orientations to self-awareness and citizenship that were highlighted in the previous section by drawing on ‘pedagogies of difference’ [19]. The ‘cloak of invisibility’ [80] that shrouds the realities of pupils from lower income and/ or immigrant families – and also shrouds the privileges that well off families take for granted -- has to be lifted within the classroom.

When this cloak does get removed at present, this often happens under circumstances that expose what are seen as vulnerabilities and that turn on a spotlight of visibility that is experienced as mortifying (sleeping on the living room sofa and not a separate bedroom in a family with limited housing, or drinking fizzy beverages where clean water is not easily available, or putting a readymade meal in the microwave oven because parents are working overtime to meet financial necessities). Teachers are therefore advised not to discuss in the classroom or elsewhere issues that are too personal from pupils‘viewpoints and that might lead to humiliation in front of classmates – instead, teachers should maintain ethical boundaries that protect pupils from too much self-disclosure [7].

However, in learning environments where pedagogies of difference and uncertainty are in use, and the multiple identities of both pupils and teachers are fully recognized and built on, it may be possible to establish “trusting learning environments where risk taking by students and teachers alike is valued and protected” ([82], p. 30–31). In this way, progress may become possible from pedagogies of invisibility or exposure, through pedagogies of protection, to pedagogies of openness within a secure environment. Further progress towards identifying factors that bulwark the political economy of health inequalities may then take place, with affluent pupils feeling able to express uneasiness with their unfair advantages. Teachers can in this way “engage their students in examining social constructions of privilege and structural inequalities and *how* these impact opportunities for students and their parents” ([82], p. 31).

Further, an ethical tone requires interaction, “meaning students are not only accountable to the teacher but also to fellow students: just because it’s your turn to talk doesn’t mean you can say whatever you want... The student must build on what somebody before has said; he or she must respond, must offer counterargument, new data, and cogent commentary… [and] accountability of performance and interaction” ([80], p. 22). In short, pupils should engage with each other as fellow citizens within civil society, and not only as classmates.

## Conclusion - re-envisioning health literacy in schools

Our arguments represent one attempt to illuminate ethical perspectives on health literacy, without claims to span all relevant perspectives. We trust that readers have been challenged to reflect on these neglected issues, as applied both to schools and to the societies that schools are embedded in.

In closing, we reiterate the need for ethical questions to be consciously and systematically addressed from early on, beginning with intentions to promote health literacy even before these intentions are translated into action, within the political space where education meets public health and health promotion. We underline again the context of fluidity and dynamism, as new challenges emerge within pedagogies and curricula, especially in response to changing populations in the society around.
